# Leaving Hospital: A Step too Far for Risk-Based Regulation?

**DOI:** 10.1093/medlaw/fwaa015

**Published:** 2020-10-21

**Authors:** Victoria L Moore

**Affiliations:** NIHR Greater Manchester Patient Safety Translational Research Centre, The University of Manchester, Manchester Academic Health Science Centre, Manchester, UK

**Keywords:** Harm, Hospital discharges, NHS, Patient safety, Risk, Risk-based regulation

## Abstract

Discharges from hospital are internationally recognised as a dangerous time in the care pathway of a patient, posing a risk to both their physical wellbeing and dignity. This article examines the effectiveness of risk-based regulation as a tool to address patient safety incidents linked to the hospital discharge process within the English National Health Service. It examines how the risk of this process is identified, conceptualised, and prioritised amongst the relevant statutory regulators, and argues that the risk is neither uniformly recognised by the statutory regulators within the English NHS, nor sufficiently addressed. Professional regulators in particular appear to have a poor awareness of the risk and their role in addressing it. Until these issues are resolved, patients leaving hospitals will continue to be exposed to patient safety incidents which should be avoidable.

## I. INTRODUCTION

Patients who are discharged from hospital are widely recognised as being at an increased risk of harm.^1^ This article seeks to demonstrate how risk-based regulation, a prominent model of regulation utilised within the English NHS by statutory regulatory bodies to protect patients from harm, is ill-equipped to ensure and improve the safety (broadly conceived) of hospital discharges. Its purpose is to draw attention to the nature of this complex problem and its impact upon patient safety; it does not seek to propose a solution. It commences by considering what regulation means within the healthcare context, and why regulators need to address safety during hospital discharges. Section II examines the rationale for risk-based models within healthcare regulation, and three weaknesses that occur when the model is applied in multi-regulator environments. The third section then considers the extent to which regulators have recognised and tackled the risk posed to patient safety by hospital discharges and the fourth section explores why the risk posed to patient safety by hospital discharges might have received limited regulatory recognition, arguing that it is a consequence of the use of risk-based regulation models are used in multi-regulator environments. This article concludes that until these weaknesses are resolved, the threat posed to patients’ safety during the discharge process will remain unmitigated.

Black defines regulation as ‘the sustained and focused attempt to alter the behaviour of others according to defined standards or purposes with the intention of producing a broadly identified outcome or outcomes’.^2^ This definition has been applied to the healthcare setting by Waring et al.^3^ and is also shared by the Professional Standards Authority (PSA). The PSA is an arm’s-length body of the Department of Health, and is responsible for regulating the professional regulators (the bodies responsible for regulating health and social care professionals).^4^ It states that a regulator’s purpose is to ‘minimise harm and to seek to do so by changing individual or organisational behaviour’.^5^ Oikonomou et al. have recently offered a broader meaning which refers to healthcare regulation as, ‘the processes engaged in by institutional actors that seek to shape, monitor, control or modify activities within healthcare organisations in order to reduce the risk of patients being harmed during their care’^6^. This definition is designed to capture the breadth of actors that are engaged in these processes, regardless of whether the actors self-identify as formal regulators. An astoundingly high number of 126 organisations were identified as exerting regulatory influence within the NHS.^7^

Although Oikonomou et al.’s broad understanding of regulation provides scope for a rich exploration of all behavioural influences, this article takes a narrower focus upon the formal attempts by statutory regulators to shape behaviour within healthcare organisations. This is because the statutory regulators have a legal duty to protect patients, and are therefore the ones held accountable for any regulatory failings that are uncovered (typically following inquiries into healthcare scandals). Given this, the regulatory bodies under consideration within this article are the professional regulators^8^; the Care Quality Commission (CQC), which regulates health and social care services; and NHS England and NHS Improvement.^9^ The latter two have regulatory oversight of healthcare services^10^ and are accountable to Parliament.^11^

The professional regulators each have the same primary purpose, established through legislation,^12^ of protecting the public. Each professional regulator shares the following overarching functions^13^: set the standards of behaviour, competence, and education that professionals must meet; address concerns raised about professionals who are unfit to practise because of poor health, misconduct or poor performance; maintain registers of professionals who are fit to practise; and set the requirements for re-registration and/or revalidation for each profession. The CQC^14^ is responsible for regulating the quality of health and social care in England. All providers of adult healthcare in England are legally required to register with the CQC, which inspects and rates the quality of services from outstanding to inadequate. The CQC sets out thirteen fundamental standards of care^15^ which cover a vast array of matters such as treating patients with dignity and respect, being open and honest when things go wrong, and ensuring appropriate staff are employed to provide care. It is accountable to Parliament and the Secretary of State for Health.^16^

### A. Patient Safety and the Risk of Harm Posed by Hospital Discharges

Patient safety is an issue of both global and national concern. The World Health Organisation (WHO) estimates that globally, the occurrence of adverse events due to unsafe care is one of the ten leading causes of death and disability^17^. In order to recognise that patient safety is a pressing health priority, the WHO launched the first World Patient Safety Day in 2019, with the aim of raising public awareness and sparking worldwide action.^18^ In the same year, NHS England and NHS Improvement published the National NHS Patient Safety Strategy. The strategy aims to continuously improve patient safety by ‘maximising the things that go right and minimising the things that go wrong for people experiencing healthcare’^19^. It predicts that getting this right could save almost one thousand extra lives and £100 million in care costs each year from 2023 to 2024.^20^

In recent years, multiple bodies tasked with improving, monitoring, or advocating for patient safety have published findings highlighting the need to reduce the number of hospital discharges which result in harm to patients. For example, Healthwatch England (HE) has published three reports drawing attention to poor hospital discharges and the resulting harm to patients since 2015.^21^ In 2016, the Parliamentary and Health Service Ombudsman (PHSO) identified four key issues that, separately, can result in patient harm and thus constitute an unsafe discharge.^22^ These are: where a patient is discharged before it is clinically safe to do so; where a patient is not assessed or consulted properly prior to discharge; where relatives or carers are not informed of the discharge; or where this no appropriate support in place for patients to cope once discharged.^23^ In response to the PHSO report’s^24^ findings, the Public Administrations and Constitutional Affairs Committee (PACAC) launched an inquiry and concluded that, ‘the incidence of unsafe discharge from NHS hospitals is much too high and this is unacceptable’.^25^

A 2015 analysis of National Reporting and Learning System^26^ (NRLS) data on discharge-related safety incidents^27^ found four main categories of error. These errors were in: the quality of discharge communication; referrals to community care; medication; and providing care adjuncts, for example wound dressings and catheters. In addition, behavioural factors, such as not following protocols, and organisational factors such as a lack of coherent guidelines^28^ were common contributory factors to patients experiencing harm. The study data further showed that the harm patients experienced because of these incidents was predominantly categorised as low-level, meaning that patients experienced mild symptoms, the harm was short-term, and little or no intervention was required to resolve the harm.^29^ However, in 78 cases (13%), patients experienced moderate harm, meaning they required an intervention to resolve symptoms and may have experienced permanent or long-term harm or loss of function. There were 3 (<1%) severe cases where life-saving interventions were required and patients experienced major loss of function, and in one instance a patient died. In one severe case for example, no discharge letter was sent to the GP, meaning the patient did not receive appropriate treatment and experienced a stroke, resulting in a permanent reduction in their function.^30^

According to Waring et al.,^31^ current thinking in the patient safety field recognises that threats to patient safety stem not only from individual errors but also from more latent factors, such as the way groups work together and the design and management of work. However, the focus for this line of thinking has tended to remain within static care domains like wards or operating departments. Waring and colleagues argue that the reason hospital discharges pose a significant patient safety problem is because latent factors are even more broadly located across the wider care system, thus presenting more complex sources of risk.^32^ Their qualitative study identified consistent threats to the safety of discharged stroke and hip fracture patients. These threats included but were not limited to: direct patient harm, for example falls, medicines-related issues, and relapse; contributing factors such as follow-up care and patient education; and latent factors, such as discharge timing, referral processes, and resource constraints. The authors concluded that hospital discharge is a ‘high risk and vulnerable stage in the patient journey’.^33^ Poor discharge planning can amount to clinical negligence, as illustrated in *Esegbona v King’s College NHS Trust.* In this case the Trust was found to be negligent in its failure to inform the nursing home that the patient was discharged to about her specific care needs. It was therefore held liable for damages for the pain, suffering and loss of amenity the patient experienced leading up to her death.^34^

In addition to experiencing physical harm related to hospital discharge processes, patients’ dignity may be harmed. This article adopts Tadd et al.’s argument that treating patients with dignity comprises of:

respectful communication; respecting privacy; promoting autonomy and a sense of control; addressing basic human needs such as nutrition, elimination and personal hygiene needs in a respectful and sensitive manner; promoting inclusivity and a sense of participation by providing adequate information to aid decision-making; promoting a sense of identity; focusing on the individual and recognising human rights.^35^

In contrast, undignified care ‘renders individuals invisible, depersonalises and objectifies people, is abusive or humiliating, narrowly focused and disempowers the individual’.^36^ The 2016 Parliamentary and Health Service Ombudsman (PHSO)^37^ report provides an example of undignified care within the context of hospital discharges: 85-year-old Mrs K, who had dementia, was discharged home late at night without her family being informed. The following morning Mrs K’s daughter found her at her home, having been left with no food, drink or bedding, and unable to care for herself or get to the toilet. We can see how such an experience is likely to have left Mrs K feeling humiliated and disempowered. Indeed, research by O’Hara et al. found that patients view such non-clinical incidents as a safety incident; within the study, one of the patient-derived safety categories was ‘Compassion/dignity/privacy/respect’.^38^

By way of further example, a British Red Cross report^39^ highlighted several instances of patients being discharged from hospital before adequate home support is in place—placing the dignity and physical wellbeing of discharged patients at risk of harm. Regarding such patients, a red cross team member stated, ‘they’ve got no family, they’ve got no one and there’s no care package in place for them coming home. They [the discharge team] just ask us to go in, and we go in and we find them, they’ve either had a fall, they’re on the floor and it’s because they’ve been sent back out too soon and they get readmitted again’.^40^

This article uses the term ‘patient safety incident’ (PSIs) to refer to any unintended or unexpected incident which could have, or did, lead to the detriment of a patient’s physical wellbeing and/or dignity. This is a broader definition than NHS Improvement’s (NHSI) definition which states that a PSI is ‘any unintended or unexpected incident which could have, or did, lead to harm for one or more patients receiving healthcare’. In the latter, harm is understood to be physical in nature.^41^ This article’s definition has a dual purpose; it reflects the fact that dignity is an important concept within healthcare from a legal and regulatory perspective, and it captures the patient perspective of harm mentioned above.

Regarding the importance of dignity from a legal perspective, the NHS Constitution, which is enshrined in the 2009 Health Act, sets out that patients have a right to be treated with dignity and respect, in accordance with their human rights.^42^ This is further stated in Regulation 10 of the Health and Social Care Act 2008 (Regulated Activities) Regulations 2014. The importance of respecting a patient’s dignity is also reflected within the professionals’ codes and the CQC’s fundamental standards.^43^ A failure to follow these standards may result in regulatory action.^44^ It is clear that regulators recognise respect for a patient’s dignity as an integral part of good health care, and thus ought to be prepared to take action to safeguard against harm to a patient’s dignity. A report by the Commission on Dignity in Care for Older People recommended that regulators must place as much emphasis on dignity in care as on clinical outcomes, and that professional regulators such as the General Medical Council (GMC) ‘must promote and enforce high standards of dignified care’.^45^

Despite the emphasis on healthcare professionals respecting patients’ dignity, the concept is not defined by the professional codes; indeed there is no clear consensus of dignity either within healthcare literature or wider philosophical literature.^46^ Alongside leaving healthcare professionals lacking practical guidance with regards to protecting patients’ dignity,^47^ the nebulous nature of dignity is problematic from a regulatory point of view. Caulfield and Brownsword argue that, given this vagueness, a requirement to respect human dignity fails to provide a clear steer to regulatees, making it difficult for regulatees to demonstrate regulatory compliance and for regulators to enforce it.^48^

This section has thus far established that patients are exposed to a risk of harm to both their physical wellbeing and their dignity during the discharge process, and argues that harm to either of these aspects ought to be of regulatory interest.^49^ The following section examines the rationale for risk-based models of regulation, and three recognised weaknesses that occur when the model is applied in multi-regulator environments.

## II. RISK-BASED REGULATION WITHIN THE ENGLISH NHS

The risk-based regulation model is intended to focus regulators’ interventions upon the threats which pose the greatest risk to its objectives, as opposed to aiming to prevent *all* possible harm.^50^ Prioritising regulatory interventions in this manner is said to be effective and proportionate,^51^ whereas to do otherwise has been called grossly inefficient.^52^ In the UK, the 2005 Hampton Report^53^ strongly endorsed risk-based approaches—describing them as essential for efficiently directing regulatory resources to where they can have maximum impact upon outcomes, and warning that a failure to use risk assessments effectively means resources may not be targeted at the riskiest areas.

Although it has been argued that a concrete definition of risk-based regulation is ‘elusive’,^54^ Black and Baldwin observe that risk-based frameworks typically take the identification of risk as their starting point, and feature the following elements^55^: a determination by the organisation as to what the risk is that it aims to control; a determination of the organisation’s ‘risk appetite’ (the type and level of risk that will be tolerated); an assessment of the likelihood of the risk occurring; and a ranking of risks based upon these assessments. In theory, these frameworks then provide a means for linking appropriate regulatory interventions to the severity of the risk.^56^ For example, in the healthcare context, a 2017 GMC Chief Operating Officer Report,^57^ illustrates the type of risk register utilised by the GMC. The register identifies not effectively sharing information as an active risk that could in turn pose a risk to patient safety. This risk was assessed as being quite likely to occur, and having a moderate impact if it did occur. Existing actions to mitigate the risk were noted, and future mitigating actions were also outlined.^58^ Similarly, the NMC’s risk register is publicly available, and identifies high-level risks, contributory factors, risk appetite, and existing and future controls.^59^

Risk can be characterised as the possibility of an undesirable incident occurring, either as a result of natural events or human activities, or due to a combination of both.^60^ Given that within healthcare, a core purpose of regulation is to minimise harm to patients, the possibility that patients will be harmed is an undesirable occurrence; that is to say it is a risk that regulators wish to reduce the occurrence of.

This article identifies three broad categories of risk that, from a regulatory perspective, could ultimately result in harm to patients. These categories are: risks to the safety and/or dignity of individual patients, risks to the reputation of regulators, and risks to the public’s trust in the healthcare professions—each of which will now be considered in turn.

The first of these, risks to the safety of individual patients, comprises of two further subcategories of risk: those that pose a risk to the physical wellbeing of a patient, and those that pose a risk to a patient’s dignity as defined above. The former is an inherent risk^61^ within the delivery of healthcare, and thus the question becomes, what level of risk is to be tolerated? For example, general anaesthesia for surgical procedures in a reasonably healthy person poses a small risk to life (approximately 1 death per 100,000 general anaesthetics^62^), but it is often recommended and accepted as part of the treatment to avoid conditions which pose a greater risk to life—such as a burst appendix. Painkillers such as tramadol also carry a risk of unpleasant side effects, ranging for example from headaches, nausea and constipation to breathing difficulties, hallucinations, and seizures.^63^ Yet for patients these risks may often be preferable to no treatment. Patients’ views of acceptable risk will also vary from patient to patient, depending upon their personal circumstances, and doctors must explore these factors with patients when discussing treatment options.^64^ On a broader level, Beaussier et al. argue that there is perpetual disagreement amongst regulators, the public and politicians, as to what constitutes acceptable risk. They note that even if agreement could be reached on what risks are acceptable at least in theory, adverse outcomes would rarely be regarded as such once they came to light.^65^

By contrast, a risk to the dignity of patients is not an inherent risk within the delivery of healthcare. This view is supported by the inquiry into poor care at Mid Staffordshire hospital, which remarked, ‘a scrutineer might reasonably have expected dignity and respect to be accorded to everyone at all times’.^66^ It is therefore argued here that the acceptable level of harm to a patient’s dignity is zero, and any risk to patient dignity, such as the hospital discharge process, should be effectively managed to obviate the chances of this risk materialising.

The second category of risk, which is harm to the reputation of a regulator, can be illustrated by a recent GMC decision to appeal a determination by the Medical Practitioners Tribunal Service (MPTS) and to call for the removal of a paediatrician from the medical register.^67^ This caused widespread outrage amongst the medical profession. In a letter addressed to the Chair of the GMC by a director of a hospital trust, the GMC was accused of undermining patient care by ‘endorsing and promoting a blame ethos that is inimical to safety’.^68^ A blame ethos poses a risk to candour which can lead to errors being hidden rather than learned from—jeopardising future patient safety.^69^ Following the aforementioned MPTS decision, the GMC commissioned an independent review into gross negligence manslaughter. The review found that doctors’ trust in the GMC had been severely damaged and that this was of great concern. It asserted that the GMC can only support doctors to deliver good medical practice if doctors feel able to engage constructively with the regulator, and have confidence that processes will ‘be proportionate, fair and just’.^70^ This is further supported by research demonstrating that regulatees respond positively to respectful, supportive approaches,^71^ and are more inclined to accept outcomes which might not otherwise appear to be in their interests if they feel they have been treated fairly.^72^ If regulatees trust the regulator, then compliance with regulatory requirements increases.^73^ Where healthcare providers’ lack trust in regulators, the quality of care provided to patients suffers.^74^

The third category of risk is to the public’s trust; Quick states that people, processes, and places within healthcare are regulated in order to ensure trust and to improve safety.^75^ Ensuring public trust in healthcare professions is central to making sure that people will seek out the healthcare that they need.^76^ High profile regulatory failures^77^ can damage the public’s trust in the ability of regulators to protect them and in the professions that they regulate.^78^

The impact of damaged trust on patient safety is illustrated in a statement by one of Dr Fata’s victims. Dr Fata was a Detroit doctor sentenced to 45 years in prison for providing medically unnecessary chemotherapy to patients.^79^ One victim stated:

‘I don’t trust any doctor or medical professional, I doubt everything they say. When I start thinking about it I can’t function, I become so anxious I can’t even go to work, and if I have a doctor’s appointment for myself or my son I cancel it. I thought it would get better with time, but it hasn’t. How am I supposed to go through the rest of my life not trusting the medical profession?^80^’

This demonstrates how a loss of trust in one medical professional can impact upon trust in the collective and ultimately threaten the safety of patients who may avoid seeking much-needed future healthcare for themselves and their children.

Risk-based regulation is intended to enable regulators to identify the risks which fall into these categories, and to determine which pose the greatest threat to their regulatory objective: minimising harm to patients. A failure to manage any of the above three categories of risk appropriately can lead to patient safety incidents. Drawing on Brownsword’s arguments,^81^ Farrell states that when measuring the effectiveness of risk-based regulation regimes, one of the elements that it is essential to consider is whether the use of the regime has enabled its aims and objectives to be met. This measurement sits alongside others: the regime’s comprehensiveness in handling risk-based issues, the extent of support or resistance afforded to it by regulatees, and the accountability mechanisms in place for monitoring it.^82^ It is upon the first of these measurements where this article focusses its attention.

### B. Identifying, Conceptualising and Prioritising Risk in Healthcare

The efficacy of risk-based regulatory approaches is heavily dependent upon successful risk identification and prioritisation. Needless to say, where the risk is unrecognised, the issue will fail to even make it upon regulators’ agenda.^83^ Black broadly sums up the difficulties of identifying risks as: ‘selecting the appropriate indicators, gathering sufficient information with respect to those indicators, assessing probabilities (particularly for low-probability, high-impact events), assessing the ability of management systems and processes to mitigate risk, and dealing with uncertainties rather than risks that can be easily calculated’.^84^

Beaussier et al. make a similar observation; that within the healthcare context regulators have struggled to assess risks to quality, to identify providers at greatest risk of failing to meet quality standards, and to prioritise inspections accordingly. This is in part because of the challenges in interpreting vast quantities of data, in devising useful indicators to capture the desired outcomes, and in making ‘credible inspection judgements about complex health organisations’.^85^ A recent inquiry^86^ by the Joint Committee on Human Rights into the detention of young people with learning disabilities and/or autism within healthcare settings illustrates the CQC’s failing in this regard. Analysis of the information available to the CQC on twenty services was examined, and a key criticism was the ‘lack of an obvious relationship between the information that CQC has available to it about a service and its inspection ratings or regulatory actions relating to that service’.^87^ The analysis found that beyond routine inspections, there appeared to be ‘little relationship between the information presented in the analysis and the timing of inspections’.^88^ The inquiry therefore concluded that the CQC was failing to meet its 2016–2021 strategic priority of delivering an ‘intelligence-driven approach to regulation’^89^ and that substantive reform of the CQC’s approach and processes is needed. This failure highlights how the challenges associated with gathering and interpreting data can lead to an inability to appropriately identify a risk to patient safety.

When risks are recognised, the priority they are subsequently afforded by regulators will be influenced by how the risk is conceptualised, as well as operational factors, and political/reputational influences.^90^ The conceptualisation of risk is particularly problematic where multiple regulators are acting within the same area.^91^ Baldwin and Black demonstrate how this is problematic within environmental regulation. A chemical used by farmers in sheep-dips potentially affects the quality of watercourses and groundwater; a risk which can be conceptualised three-ways: as a harm to the environment, a harm to animal health, and a harm to human health. Each of these harms may be the responsibility of more than one regulator, and subject to differing legal regimes. By way of further example, in 2018 the Health and Social Committee published a report^92^ regarding how NHS Digital was sharing confidential patient information with the Home Office to trace immigrants. Information included patients’ names, date of birth, last known address and their GP’s contact details. NHS Digital had argued that sharing this information was within the public interest because it enabled the effective enforcement of immigration law. This allegedly outweighed concerns that it might impact broader public trust in a confidential health service.^93^ Several bodies, including the GMC, British Medical Association (BMA), Public Health England (PHE) argued that this information sharing posed a serious risk to public health, risked undermining public trust in a confidential health service, and placed doctors at risk of failing to comply with their professional guidance.^94^

The above demonstrates how different organisations with regulatory influence conceptualise risk. Whereas the risk to public trust in the health service was seen to be low by NHS Digital, it was seen as a high risk by the GMC. Differing conceptualisations of risk such as this can result in different levels of priority being assigned to the risk, resulting in poor regulatory coordination and effectiveness.^95^

Black and Baldwin argue that in reality, a risk that is categorised as ‘low’ equates to ‘low priority’^96^ for regulators, and is a statement of the risk’s relative significance to the regulator and their potential to meet their objectives.^97^ They provide a comprehensive overview of how risks that are classified as low can have the potential to cause significant harm, and as such still require regulatory attention.^98^ For example, within the context of water quality regulation, an individual farm engaging in an activity such as the cleaning of milking parlours might be seen to only pose a low risk to water quality, because only small quantities of effluent are discharged into water sources during the cleaning process. However, when such activities which present a low risk at an individual site are engaged in by the masses, the risk may accumulate to become systemic.^99^ Likewise, an individual clinician’s poor handwashing technique may only pose a low risk to the overall safety of the entire patient population, but when practised by multiple clinicians would pose a high risk to public health.

This section has introduced the model of risk-based regulation used by healthcare regulators, and argued that harm to patients is the primary risk that regulators seek to manage. It has identified three broad categories of risk that can result in harm to patients. These are: direct risks of harm to the physical wellbeing and dignity of patients; risks to a regulator’s reputation; and risks to public trust in the professions. It has then examined how the identification of these risks can be challenging for regulators, and how incohesive conceptualisations of risks and their subsequent prioritisation by multiple regulators informs their regulatory response. Against this backdrop, the following section examines the extent to which statutory healthcare regulators have recognised and tackled the risk posed to patient safety by hospital discharges.

## III. REGULATORY REACTION TO THE HOSPITAL DISCHARGE SAFETY RISK

As discussed earlier, in 2016 the Public Administrations and Constitutional Affairs concluded that the incidence of unsafe hospital discharges was unacceptably high,^100^ and Healthwatch England (HE) have repeatedly drawn attention^101^ to the harm patients are exposed to when leaving hospital. The focus here is on whether the risk posed by hospital discharges is recognised by statutory healthcare regulators within the English NHS.

The CQC’s assessment framework,^102^ which reflects its five core questions when inspecting healthcare services,^103^ indicates that the risk posed by hospital discharges to patient safety is a risk they are aware of. The framework contains a number of Key Lines of Enquiry (KLOEs), three of which feature questions relating to the safety, effectiveness, and responsiveness of hospital discharges. It asks firstly whether all the information needed for a patient’s ongoing care is shared appropriately, in a timely way and in line with relevant protocols at the point of discharge.^104^ Secondly, it asks if all relevant teams, services, and organisations are informed when people are discharged from a service, and if discharge is undertaken at an appropriate time of day and only when necessary ongoing care is in place.^105^ Thirdly, the framework asks how people are supported during discharge.^106^ Information provided in response to a freedom of information request^107^ stated that breaches in relation to safe discharge are most likely to be under Regulation 9 of the Health and Social Care Act 2008 (Regulated Activities) Regulations 2014, which is ‘person-centred care’. This regulation is designed to ensure that people using a service have care or treatment that is personalised for them. The regulation guidance states: ‘assessments should be reviewed regularly and whenever needed throughout the person’s care and treatment. This includes when they transfer between services, use respite care or are re-admitted or discharged. Reviews should make sure that people’s goals or plans are being met and are still relevant’.^108^ If the CQC finds that a provider is in breach of this regulation it can use its regulatory powers to require or force a provider to improve.^109^

The CQC’s 2018 adult inpatient survey report highlighted hospital discharge planning as an area for improvement. It flagged that 44% of respondents discharged with medication were not being told about possible side-effects to watch out for, and only one in four were being told who to contact if they were worried about their condition following discharge. Seventeen per cent of respondents commented they felt uninvolved in their discharge planning—an area which has seen no improvements in 10 years.^110^

The CQC also has an ‘independent voice’ role, under which a range of reports regarding quality and safety of services are published; for example in 2019 the CQC published a report^111^ on medicines optimisation which included two recommendations for safe discharge—essentially highlighting the importance of relevant and timely information sharing between hospitals and other services following discharge.^112^ Furthermore, HE is a statutory committee of the CQC whose purpose includes escalating concerns raised by local Healthwatch organisations to the CQC.^113^ As mentioned earlier in this article, HE has published three reports regarding unsafe hospital discharges.^114^ Thus it seems reasonable to conclude that the risk to patients posed by hospital discharges is a risk that is known to the CQC.

In 2014, NHSE issued a patient safety alert to NHS organisations stating that approximately 33% of 10,000 incidents reported to the National Reporting and Learning System (NRLS) between October 2012 and September 2013 involved patients discharged from hospital without sufficient and timely communication of essential information. In some instances, this led to ‘avoidable death and serious harm to patients due to a failure in continuity of care as well as avoidable readmission to secondary care’.^115^ NHSI publishes online resources in order to support the safe discharge of patients throughout the NHS,^116^ an aim NHSI acknowledged responsibility for when giving evidence to the PACAC committee regarding unsafe hospital discharges.^117^ Once again it seems reasonable to conclude that the risk to patients posed by hospital discharges is known to NHSI/NHSE.

In contrast to the above organisational regulators, it is not immediately apparent that the risk posed by hospital discharge is recognised by the professional regulators. None of the professional regulators gave evidence to the PHSO inquiry regarding unsafe discharges^118^ and none of the professional codes that registrants are expected to follow specifically mention discharges. Although discharges are not directly referred to, each code^119^ does include the core behaviours and skills which are essential for ensuring safe discharge, such as: good communication with patients and colleagues; competency; multi-disciplinary working; safe prescribing; record-keeping; continuity of care; and the importance of working in partnership with patients. However, there is little evidence regarding how, if at all, professional codes positively influence behaviour.^120^ As such, it cannot be argued that these professional codes, by themselves, are a sufficient regulatory response to the patient safety posed by hospital discharges.

Moreover, recent British Red Cross^121^ research into safe hospital discharges did not include any interviews with professional regulators, which may suggest that the risk is perceived as belonging to the systems regulators rather than the professional regulators. It is worth emphasising again at this point that patient safety incidents linked to the hospital discharge process *is* an issue that professional regulators should be interested in. In January 2020, the Guardian reported that the Royal Cornwall Hospitals NHS Trust had informed staff that patients should be discharged early to reduce overcrowding; a risk it called ‘proportionate’ despite the possibility ‘that some of these patients will be readmitted or possibly come to harm’.^122^ To require clinicians to act in such a manner is asking them to act in a way which may go against the professional standards expected of them, such as making the care of their patients their first concern and providing dignified care.^123^ This is something which professional regulators should address.

Returning to whether professional regulators are aware of the discharge risk—a publicly available update on the GMC’s harms reduction programme^124^ in 2018 briefly mentions hospital discharges as a potential cause of harm. The document states that the purpose of the harms reduction programme is to support doctors to maintain good medical practice by ‘identifying, understanding and addressing problems that might impede the delivery of this and by extension, present a risk of harm to patients or doctors’.^125^ The draft harms register within the document identifies as a potential harm for future consideration ‘inappropriate discharge’ such as ‘individuals being discharged prior to the results of investigations – particularly in A&E’.^126^ This harm is categorised as a ‘process failure/non-compliance’ issue within a broader category of ‘system-level harms’.^127^

This section has thus far established that although the harm of hospital discharges is recognised by the CQC, NHSE, and NHSI, it is not widely recognised or acknowledged amongst the professional regulators, at least within the public sphere. This raises a further pertinent question: why might the risk posed to patient safety by hospital discharges be missing from the professional regulators’ agenda? It is to this question that we now turn.

## IV. IDENTIFYING, CONCEPTUALISING, AND PRIORITISING THE RISK OF HOSPITAL DISCHARGES

In order to answer the question of why this risk might not be recognised by the professional regulators, it is apposite to return to the difficulties discussed in Section III concerning the identification, conceptualisation, and prioritisation of risk.

### A. Identification of Risk

The first reason that the risk may be unrecognised is because the success of risk-based regulation approaches is heavily dependent upon the availability of sufficient information to inform decision-making.^128^ Given the web of actors within the English NHS, and the mass of information held amongst them, an individual actor is unlikely to possess all of the relevant information it would need to react accordingly.^129^ For example, professional regulators have historically relied heavily upon complaints made by patients, their families or employers about an individual healthcare professional to trigger an investigation into the individual’s fitness to practice (FTP). A risk-based approach to assessing the risk of harm to patients posed by an individual is then typically followed, which allows regulators to justify their decision-making processes.^130^ However, professional regulators are adopting a more ‘upstream’ approach to regulation. This means they are moving towards ‘pro-active, early and specific interventions in order to either decrease the likelihood of an undesirable outcome or to increase the likelihood of a more favourable outcome’.^131^ Complaints about individual practitioners alone are an insufficient data source for identifying and addressing broader, complex safety issues such as hospital discharges, where patient safety is not dependent upon the actions of an individual.

Positive steps have been taken to address this information deficit. For example, the GMC’s health system liaison service was created to help the GMC engage at every level with the healthcare systems, helping to ensure that their approach to regulation is well informed.^132^ The service sees GMC advisers collaborate with doctors, educators, employers, and other regulators in order to ‘understand, identify and address risks to patients and doctors before harm occurs’.^133^ The GMC’s corporate risk register^134^ also outlines some of its existing mechanisms for sharing data. For example, the register states that the GMC works closely with the Health and Social Care Regulators’ Forum to improve collaboration, holds regular surveillance groups with the CQC to consider risk, has regular intelligence sharing meetings called Regional Information Forums, engages with NHS Improvement, and has a central analytics team in place which is responsible for coordinating data sharing. Hospital discharges are specifically mentioned in a memorandum of understanding (MoU) in place between the CQC and GMC.^135^ This states that the CQC would like to be informed of issues affecting patient experience, including delays in discharge, early discharge, and lack of dignity or respect to patients.^136^ The GMC indicated they would wish to be informed of scenarios such as foundation doctors in surgery signing discharge letters that have been written by other doctors relating to patients they have never examined.^137^

Furthermore, in 2018, an emerging concerns protocol^138^ was developed amongst regulators, which five of the professional regulators^139^ have signed. The protocol is designed to establish a method for sharing early concerns so that links between concerns can be made. The concerns may fall into the following three categories: ‘concerns about individual or groups of professionals; concerns about healthcare systems and the healthcare environment (including the learning environments of professionals); concerns that might have an impact on trust and confidence in professionals or the professions overall’.^140^

Clearly apparent above is the sheer quantity of mechanisms in place in order to facilitate information sharing across regulators, yet despite these a recent inquiry has concluded that there is an ‘insufficient linkage between CQC and the other regulators’^141^. Due to the vast amounts of data that each regulator is likely to hold, it is highly unlikely that all issues will be shared amongst all regulators. In practice, judgment calls will need to be made about what issues are shared across which forum at any given time. It is therefore possible that so far, information relating to PSIs within the context of hospital discharges has not been widely shared and considered amongst all of the healthcare regulators—leading to poor identification of the risk posed to patients.

### B. Conceptualisation of Risk

This leads to the second reason why the risk posed by hospital discharges might be missing from the professional regulators’ agenda: the challenge of conceptualising risk in a unified manner where multiple regulators^142^ are involved. How each regulator constructs the risk posed by hospital discharges will determine *if and how* the information is shared across the regulators.

As established in Section II, from a regulatory perspective there are three broad categories of risk that can result in harm to patients: risks to the physical wellbeing/and or dignity to the patient; risks to a regulator’s reputation; and risks to public trust in the professions. The risk posed by hospital discharges is likely to fall predominantly within the first category; however, professional regulators may still not conceptualise it as a risk within their remit. For example, the GMC’s harm register indicates that the GMC perceives inappropriate discharge as a process failure/non-compliance issue, listed under a broader heading of system level harm.^143^ Construed in this manner, it may not be apparent that this is also a risk closely entwined with the behaviour of healthcare professionals, including doctors. Yet as the scenario of Mrs K highlighted, the decision to discharge Mrs K in the given circumstances was not in line with behavioural expectations set out in any of the professional codes, and resulted in harm to her dignity; which is a patient safety incident.

Within the Cornwall^144^ example, we can see how the risk is situated not only within the remit of the systems regulators (the pressure of under-resourced hospitals), but also within the remit of the professional regulators. This is because the situation is likely to impact upon the ability of healthcare professionals to act in accordance with their professional standards – for releasing patients before they are clinically ready is unlikely to be cohesive with providing good care for a patient. This particular case straddles two of the categories of risk identified in Section II; direct risk to a patient’s physical wellbeing or dignity, and risk to public trust in the professions.

By way of further example, the MoU between the GMC and CQC^145^ shows the GMC has an interest in receiving information from the CQC regarding foundation doctors signing discharge letters that have been written by other doctors and relating to patients they have never examined. This is the only discharge-specific scenario that the GMC provides as an example of the type of discharge-related issue it is interested in. The rationale for this interest is that it might cause a patient safety concern or indicate bullying concerns.^146^ However, by requesting such specific information on discharges, the GMC may inadvertently be signalling that it is not interested in being informed of discharge-related harms to a patient’s dignity, despite the fact that respect for patient dignity is a central feature of the GMC’s expectations of doctors.^147^

### C. Prioritisation of Risk

Thirdly, the risk posed by hospital discharges could be missing from the professional regulators’ agenda due to being categorised as low risk, and thus as Black argues, low priority.^148^ One reason why the risk might be categorised as ‘low’ is that the resulting physical harm is typically mild (Williams’ study^149^ of NRLS data reported 64% of discharge-related harm was low-level). Thus, if activities are categorised according to the severity of physical harm, then hospital discharges may not be perceived as being high-risk. Risks that catch the attention of the media and dominate headlines are also more likely to be treated as a higher priority due to the threat posed to the reputation of the regulator and to public trust in the profession. To-date, the harm posed by hospital discharges to patient safety has had limited recognition^150^ in the media, despite the ongoing nature of the problem.

The frequency of harm to dignity is harder to measure than physical harm, partly because harm to dignity is likely to go unreported. Research by the PHSO has highlighted that despite being the greatest users of health and social care providers (thus subject to frequent discharges), older people are reluctant to complain about poor care.^151^ However, as discussed in Section I of this article, harm to dignity is a patient safety concern arising during hospital discharges. Given that all patients have a right^152^ to be treated with dignity, this is not a harm that should be ignored or categorised as low-priority by professional regulators.

This section has examined why the professional regulators may not have adequately addressed the risk posed to patient safety by hospital discharges. However, it is important to note that responsibility for responding to this particular patient safety risk does not lie solely with the professional regulators; a cohesive response from all of the statutory regulators is required. The first step in achieving this is to overcome the challenges laid out in this article regarding the identification, conceptualisation, and prioritisation of this patient safety risk.

## V. CONCLUSION

This article has highlighted the risk posed to patient safety by the hospital discharge process. It has examined the nature of the risk of harm patients face during discharge; namely harm to the physical wellbeing and to their dignity. It has then identified the regulators who ought to be reducing the risk of such harm to patients, and highlighted the minimal actions that have been taken to achieve this aim. In order to establish why there has been a lack of regulatory action in this area, particularly from the professional regulators, this article has considered the risk-based regulation model which is utilised by these regulators.

Consideration of this model has focussed upon three weaknesses regarding how regulators identify, conceptualise, and subsequently prioritise risk. The difficulties regulators face with these three elements has meant regulatory interventions to ensure patient safety during hospital discharge have been limited.

In the case of hospital discharges, the first difficulty regarding risk-identification arises as regulators do not possess a holistic overview of all relevant information. This is because of the multitude of regulators and the limited information-sharing mechanisms between them—which means judgements have to be made about what information to share and with whom. This is problematic given that successful risk-based regulation is heavily dependent upon the availability of sufficient information to identify risks and inform decision-making.

The multitude of statutory regulators and limited information-sharing leads to a further difficulty: it is virtually impossible for them all to have a unified understanding of the risk posed by discharges. Risks will be conceptualised based upon the nature of information possessed, which will vary in a field saturated with regulators.

Finally, successful risk-based regulation relies upon the correct prioritisation of risk, an outcome which is reliant upon regulators having obtained sufficient information and having clarity amongst themselves regarding their regulatory aim. It is possible that regulators are not prioritising ensuring patient safety during discharge in the manner they would if they had the requisite information and clarity about the risk that discharges pose to patients.

Combined, these three weaknesses have meant that the risk posed to patient safety at the point they leave hospital is neither uniformly recognised by the statutory regulators within the English NHS, nor sufficiently addressed. Professional regulators in particular appear to have a poor awareness of the risk and their role in addressing it. The result of this ineffective regulation leaves the physical wellbeing and dignity of patients continuously imperilled at a point in time when they should be returning safely home. Until regulators can accurately identify this risk, build a unified understanding of its causes and consequences, and prioritise it appropriately, this unacceptable status quo will remain.

## Footnotes

1 See World Health Organisation, *Transitions of Care: Technical Series on Safer Primary Care* (World Health Organisation 2016); K Aase and others, *Researching Quality in Care Transitions International Perspectives* (1st edn, ebook, Palgrave Macmillan 2017); K Manges and others, ‘A Mixed Methods Study Examining Teamwork Shared Mental Models of Interprofessional Teams during Hospital Discharge’ (2019) BMJ Quality & Safety.

2 J Black, ‘Decentering Regulation: Understanding the Role of Regulation and Self-regulation in a Post-regulatory World’ (2001) 54 Current Legal Problems 142.

3 J Waring and others, ‘Modernising Medical Regulation: Where are We Now’ (2010) 24 Journal of Health Organisation and Management 6, 541.

4 For further information on the PSA holds professional regulators accountable see J Allsop and K Jones, ‘Regulating the Regulators: The Rise of the United Kingdom Professional Standards Authority’ in J Chamberlain, M Dent and M Saks (eds), *Professional Health Regulation in the Public Interest: International Perspectives* (1st edn, Policy Press 2018) and O Quick, *Regulating Patient Safety* (1st edn, CUP 2017).

5 Professional Standards Authority, *Rethinking Regulation* (PSA 2015).

6 E Oikonomou and others, ‘Patient Safety Regulation in the NHS: Mapping the Regulatory Landscape of Healthcare’ (2019) 9 BMJ Open 2.

7 ibid 1–9.

8 General Medical Council, General Dental Council, General Chiropractic Council, General Optical Council, General Osteopathic Council, General Pharmaceutical Council, Health and Care Professions Council, Nursing and Midwifery Council, and Social Work England.

9 Since April 2019 these have been working together as a single organisation. See NHS Improvement, ‘Working Together’ (2019) <https://improvement.nhs.uk/> accessed 12 June 2020.

10 For further detail on differences in responsibilities between CQC and NHSI see: British Medical Association, ‘The Regulatory Systems for Healthcare Quality across the United Kingdom’ (2016) <https://www.bma.org.uk/collective-voice/policy-and-research/nhs-structure-and-delivery/monitoring-quality-in-the-nhs/regulatory-systems-for-healthcare-quality> accessed 12 June 2020.

11 NHS England, ‘Accountability Report’ (2019) <https://www.england.nhs.uk/wp-content/uploads/2019/07/accountability-report-201819.pdf> accessed 12 June 2020.

12 See the Medical Act 1983, Dentists Act 1984, Chiropractors Act 1994, Opticians Act 1989, The Osteopaths Act 1993, The Health Act 1999, the Nursing and Midwifery Order 2001, The Health and Social Work Professions Order 2001, and the Pharmacy Order 2010.

13 Law Commission, *Regulation of Health Care Professionals: Regulation of Social Care Professionals in England* (Law Commission 2014).

14 Health and Social Care Act 2008, s 3(1)–(2).

15 Care Quality Commission, 'The Fundamental Standards’ (2019) <https://www.cqc.org.uk/what-we-do/how-we-do-our-job/fundamental-standards> accessed 12 June 2020.

16 Care Quality Commission, ‘Framework Agreement between the Department of Health and Care Quality Commission’ (2013). <https://www.cqc.org.uk/sites/default/files/documents/cm_0114310_item_10_appendix_1_cqc_framework_agreement.pdf> accessed 12 June 2020.

17 World Health Organisation, ‘Patient Safety’ (2019) <https://www.who.int/news-room/fact-sheets/detail/patient-safety> accessed 12 June 2020.

18 World Health Organisation, ‘World Patient Safety Day’ (2019) <https://www.who.int/campaigns/world-patient-safety-day/2019> accessed 12 June 2020.

19 NHS England and NHS Improvement, ‘The NHS Patient Safety Strategy: Safer Culture, Safer Systems, Safer Patients’ (2019) <https://improvement.nhs.uk/documents/5472/190708_Patient_Safety_Strategy_for_website_v4.pdf> accessed 12 June 2020 6.

20 ibid.

21 Healthwatch England, *Safely Home: What Happens when People Leave Hospital and care settings?*‘ (Healthwatch 2015); Healthwatch England, ‘What Happens when People Leave Hospital and other Care Settings?’ (Healthwatch 2017); Healthwatch England, ’Emergency Readmissions: What’s Changed One Year On?' (Healthwatch 2018).

22 Parliamentary and Health Service Ombudsman, ‘A Report of Investigations into Unsafe Discharge from Hospital’ (PHSO 2016).

23 Public Administration and Constitutional Affairs Committee, ‘Follow-up to PHSO Report on Unsafe Discharge from Hospital’ (PACAC 2016).

24 Parliamentary and Health Service Ombudsman (n 23).

25 Public Administration and Constitutional Affairs Committee (n 24) 18.

26 The NRLS is a central database of patient safety incidents (PSIs) reported from across England and Wales. A PSI for the purpose of the database is defined as ‘any unintended or unexpected incident which could have, or did, lead to harm for one or more patients receiving healthcare’. See NHS Improvement, ‘Report a Patient Safety Incident’ (2017) <https://improvement.nhs.uk/resources/report-patient-safety-incident/> accessed 12 June 2020.

27 H Williams and others, ‘Harms from Discharge to Primary Care: Mixed Methods Analysis of Incident Reports’ (2015) 65 British Journal of General Practice 641, e829–e837.

28 ibid.

29 ibid.

30 ibid.

31 J Waring and others, ‘A Qualitative Study of Professional and Carer Perceptions of the Threats to Safe Hospital Discharge for Stroke and Hip Fracture Patients in the English National Health Service’ (2016) 16 BMC Health Services Research.

32 ibid.

33 ibid.

34
*Esegbona v King’s College NHS Trust* [2019] EWHC 77 (QB).

35 W Tadd and others, ‘Dignity in Practice: An Exploration of the Care of Older Adults in Acute NHS Trusts’ (The Stationary Office: London 2011) 10.

36 ibid 10.

37 Parliamentary and Health Service Ombudsman (n 23).

38 JK O’Hara and others, ‘What Can Patients Tell Us About the Quality and Safety of Hospital Care? Findings From A UK Multicentre Survey Study’ (2018) 27 BMJ Quality & Safety 27, 673–682.

39 British Red Cross, ‘In and Out of Hospital’ (British Red Cross 2018).

40 ibid 11.

41 NHS Improvement, ‘Report A Patient Safety Incident’ (2017) <https://improvement.nhs.uk/resources/report-patient-safety-incident/> accessed 12 June 2020.

42 National Health Service, ‘NHS Constitution for England’ (NHS 2015).

43 For example, see para 25 of ‘Good Medical Practice' (2013); para 1 of the NMC’s Code (2015); standard 1 of ‘Standards for Pharmacy Professionals’ (2017); s 2.2 of Social Work England’s Professional Standards (2019) and the CQC’s ‘The Fundamental Standards’ (2019).

44 General Medical Council, ‘Good Medical practice’ (2013) para 6.

45 Commission on Dignity in Care for Older People, ‘Delivering Dignity: Securing Dignity in Care for Older People in Hospitals and Care Homes’ (Commission on Dignity in Care for Older People 2011) Recommendation 35.

46 L Barclay, ‘In Sickness and in Dignity: A Philosophical Account of the Meaning of Dignity in Health Care' (2016) 61 International Journal of Nursing Studies 136–141.

47 ibid.

48 T Caulfield and Brownsword R, 'Human Dignity: A Guide to Policy Making in the Biotechnology Era?’ (2006) 7 Science and Society 72–76.

49 There are of course additional emotional harms which a patient might experience, such as stress and anxiety. This article focusses on harm to dignity because of the regulatory requirements to respect patient dignity.

50 Beaussier and others (n 2) 206.

51 H Rothstein, ‘The Institutional Origins of Risk: A New Agenda for Risk Research’ (2006) 8 Health, Risk and Society 3, 215–21.

52 Beaussier and others (n 2) 206.

53 P Hampton, *Reducing Administrative Burdens: Effective Inspection And Enforcement* (HM Treasury 2005).

54 S Lloyd-Bostock and B Hutter, 'Reforming Regulation of the Medical Profession: The Risks of Risk-based Approaches’ (2008) 10 Health, Risk and Society 1, 70.

55 J Black and R Baldwin, ‘Really Responsive Risk-based Regulation’ (2010) 32 Law and Policy 2, 181–213.

56 ibid.

57 General Medical Council, ‘Chief Operating Officer’s Report: Agenda Item M4’ (2017) <https://www.gmc-uk.org/-/media/documents/m04—chief-operating-officer-s-report_pdf-72010185.pdf> accessed 12 June 2020 11.

58 ibid.

59 Nursing and Midwifery Council, ‘Council Meeting: 27 March 2019’ (2019) <https://www.nmc.org.uk/about-us/governance/the-council/council-meetings/previous-council-meetings/council-meeting-27-march-2019/> accessed 12 June 2020.

60 J Black, ‘The Role of Risk in Regulatory Processes’ in R Baldwin, M Cave and M Lodge (eds), *The Oxford Handbook of Regulation* (1st series, OUP 2010).

61 D Sohn, ‘Negligence, Genuine Error, and Litigation’ (2013) 6 International Journal of General Medicine 49–56.

62 Royal College of Anaesthetists, ‘Section 15: Death or Brain Damage’ (2017) <https://www.rcoa.ac.uk/sites/default/files/documents/2019-11/15-DeathBrainDamageweb.pdf> accessed 12 June 2020.

63 National Health Service, ‘Tramadol’ (2018) <https://www.nhs.uk/medicines/tramadol/> accessed 12 June 2020.

64
*Montgomery v Lanarkshire Health Board* [2015] SC 11 [2015] 1 AC 1430; General Medical Council, ‘Consent: Patient and Doctors Making Decision Together' (2019) <https://www.gmc-uk.org/ethical-guidance/ethical-guidance-for-doctors/consent> accessed 12 June 2020.

65 Beaussier and others (n 2).

66 R Francis, ‘ *Report of the Mid Staffordshire Foundation Trust Public Inquiry Volume I* (The Stationary Office 2013) 6.262.

67 B Gault, ‘GMC was Advised to Appeal Bawa-Garba Case to “protect reputation of profession”' (2019) <http://www.pulsetoday.co.uk/news/all-news/gmc-was-advised-to-appeal-bawa-garba-case-to-protect-reputation-of-profession/20039140.article> accessed 12 June 2020.

68 N Ross, ‘Letter to the GMC Chair Regarding Hadiza Bawa-Garba’ (2018) <https://www.bmj.com/content/360/bmj.k195> accessed 19 December 2019.

69 National Advisory Group on the Safety of Patients in England, *A Promise to Learn – A Commitment to Act: Improving the Safety of Patients in England* (Crown Publishing 2013); NHS England and NHS Improvement (n 20).

70 L Hamilton, ‘Independent Review of Gross Negligence Manslaughter and Culpable Homicide’ (2019) 22 <https://www.gmc-uk.org/-/media/documents/independent-review-of-gross-negligence-manslaughter-and-culpable-homicide—final-report_pd-78716610.pdf> accessed 12 June 2020.

71 J Healy, *Improving Healthcare Safety and Quality: Reluctant Regulators* (1st edn, Ashgate Publishing 2011).

72 TR Tyler, ‘Procedural Justice, Legitimacy, and the Effective Rule of Law’ (2003) 30 Crime and Justice 283–357.

73 J Braithewaite and T Makkai, ‘Trust and Compliance’ (1994) 4 Policing and Society 1; K Murphy, ‘The Role of Trust in Nurturing Compliance: A Study of Accused Tax Avoiders’ (2004) 28 Law and Human Behaviour 2, 187–209.

74 S Kane and others, ‘Trust and Trust Relations from the Providers’ Perspective: The Case of the Healthcare System in India’ (2015) 12 Indian Journal of Medical Ethics 3, 157–68.

75 Quick (n 5).

76 See Healy (n 72) for further discussion on the importance of trust within healthcare.

77 See eg J Smith, *The Shipman Inquiry Fifth Report: Safeguarding Patients: Lessons from the Past - Proposals for the Future* (The Stationary Office 2004); Francis (n 67); G James, *Report of the Independent Inquiry into the Issues raised by Paterson* (House of Commons 2020).

78 J Allsop, ‘Regaining Trust in Medicine: Professional and State Strategies’ (2006) 54 Current Sociology 4 621–36.

79 Justice Department, ‘Detroit Area Doctor Sentenced To 45 Years In Prison For Providing Medically Unnecessary Chemotherapy To Patients’ (2015) <https://www.justice.gov/opa/pr/detroit-area-doctor-sentenced-45-years-prison-providing-medically-unnecessary-chemotherapy> accessed 12 June 2020.

80 Justice Department, ‘Farid Fata Victim Impact Statements’ 2:13-cr-20600-PDB-DRG Doc # 135-2 (2015), 22.

81 R Brownsword, *Rights, Regulation, and The Technological Revolution* (1st edn, OUP 2008).

82 AM Farrell, *The Politics of Blood: Ethics, Innovation and the Regulation of Risk* (1st edn, CUP 2014) 199.

83 R Baldwin and J Black, ‘Driving Priorities in Risk-based Regulation: What's the Problem?’ (2016) 43 Journal of Law and Society 4, 565–95; F Haines, ‘Regulation and Risk’ in P Drahos (ed), *Regulatory Theory: Foundations and Applications* (ANU Press 2017).

84 Baldwin and Black, ibid.

85 Beaussier and others (n 2) 205–24.

86 Joint Committee on Human Rights, ‘The Detention of Young People with Learning Disabilities and/or Autism’ (House of Commons 2019).

87 ibid 45.

88 ibid 45.

89 CQC, ‘Our Strategy for 2016-2021’ (2017) <https://www.cqc.org.uk/about-us/our-strategy-plans/our-strategy-2016-2021> accessed 12 June 2020.

90 Baldwin and Black (n 84).

91 ibid.

92 House of Commons Health and Social Care Committee, ‘Memorandum of understanding on data-sharing between NHS Digital and the Home Office’ (House of Commons Health and Social Care Committee, 2018).

93 ibid.

94 House of Commons Health and Social Care Committee (n 93); General Medical Council, ‘Data-sharing Agreement could Threaten Patient Confidentiality’ (2018) <https://www.gmc-uk.org/news/news-archive/data-sharing-agreement-could-threaten-patient-confidentiality> accessed 12 June 2020.

95 Baldwin and Black (n 84).

96 J Black and R Baldwin, ‘When Risk-based Regulation Aims Low: Approaches and Challenges’ (2012) 6 Regulation and Governance 1, 4.

97 Baldwin and Black (n 84).

98 ibid 2–22.

99 ibid 2–22.

100 Public Administration and Constitutional Affairs Committee (n 24) 18.

101 See n 22.

102 Care Quality Commission, ‘Key Lines of Enquiry, Prompts and Ratings Characteristics for Healthcare Services’ (2018) <https://www.cqc.org.uk/sites/default/files/20180628%20Healthcare%20services%20KLOEs%20prompts%20and%20characteristics%20FINAL.pdf> accessed 12 June 2020.

103 These five questions ask whether services are safe, effective, caring, responsive and well led.

104 CQC KLOE S3.3: When people move between teams, services and organisations (which may include at referral, discharge, transfer and transition), is all the information needed for their ongoing care shared appropriately, in a timely way and in line with relevant protocols?

105 CQC KLOE E4.4: Are all relevant teams, services and organisations informed when people are discharged from a service? Where relevant, is discharge undertaken at an appropriate time of day and only done when any necessary ongoing care is in place?

106 CQC KLOE R2.3: How are people supported during referral, transfer between services and discharge?

107 Correspondence between author and CQC (August 2019).

108 Care Quality Commission, Guidance for providers on meeting the regulations (CQC 2015) 31.

109 Information provided to author in private correspondence with CQC (August 2019).

110 Care Quality Commission, ‘2018 Adult Inpatient Survey: Statistical release’ (CQC 2019).

111 Care Quality Commission, ‘Medicines in Health and Adult Social Care: Learning from Risks and Sharing Good Practice for Better Outcomes’ (CQC 2019).

112 ibid 35 and 52.

113 Healthwatch England, ‘Our history and functions’ <https://www.healthwatch.co.uk/our-history-and-functions> accessed 12 June 2020.

114 See n 22.

115 NHS England, ‘Patient Safety Alert NHS/PSA/W/2014/014’ (NHSE 2014) <https://www.england.nhs.uk/wp-content/uploads/2014/08/psa-imp-saf-of-discharge.pdf> accessed 12 June 2020.

116 See eg NHS Improvement, ‘A guide to developing criteria-led discharge’ (2017) <https://improvement.nhs.uk/resources/guide-developing-criteria-led-discharge/> accessed 12 June 2020; NHS Improvement, ‘Discharge planning’ (2018) <https://improvement.nhs.uk/resources/discharge-planning/> accessed 12 June 2020.

117 Public Administration and Constitutional Affairs Committee, ‘Discharging Older People from Acute Hospitals’ (PACAC 2016/17) 15.

118 Parliamentary and Health Service Ombudsman (n 23).

119 See eg General Medical Council (n 9), Nursing and Midwifery Council, ‘The Code’ (2015), General Pharmaceutical Council, ‘Standards for Pharmacy Professionals’ (2017) and Social Work England, ‘Professional Standards’ (2019).

120 O Quick, ‘A Scoping Study on the Effects of Health Professional Regulation on those Regulated’ (Council for Healthcare Regulatory Excellence 2011); Healy (n 72).

121 British Red Cross, ‘Home to the Unknown: Getting Hospital Discharge Right’ (British Red Cross 2019).

122 The Guardian, ‘Cornwall Hospital to Discharge Patients Early Despite Saying it may be Harmful’ (2020) <https://www.theguardian.com/society/2020/jan/14/cornwall-hospital-to-discharge-patients-early-despite-risks> accessed 12 June 2020.

123 General Medical Council (n 45).

124 General Medical Council, ‘Executive Board Meeting’ (2018) <https://www.gmc-uk.org/-/media/documents/08—the-harms-reduction-programme-progress-update_pdf-75445141.pdf> accessed 12 June 2020.

125 ibid 47.

126 ibid 59.

127 ibid 59.

128 Hampton (n 54); S Lloyd-Bostock and B Hutter, ‘Reforming Regulation of the Medical Profession: The Risks of Risk-based Approaches’ (2008) 10 Health, Risk and Society 1, 69–83.

129 Healy (n 72).

130 For discussion of the GMC’s FtP risk-based approach see JM Chamberlain, ‘Malpractice, Criminality, and Medical Regulation: Reforming the Role of the GMC in Fitness to Practise Panels’ (2017) 25 Medical Law Review 1, 1–22 and S Lloyd-Bostock, ‘The Creation of Risk-Related Information: The UK General Medical Council’s Electronic Database’ (2010) 24 Journal of Health Organisation and Management 6, 584–89.

131 General Medical Council (n 125) 53.

132 General Medical Council, ‘Health System Liaison Services’ <https://www.gmc-uk.org/about/how-we-work/liaison-and-outreach/health-system-liaison-services> accessed 12 June 2020.

133 ibid.

134 General Medical Council (n 45).

135 CQC and GMC, ‘Operational Protocol: A Practical Guide for Staff - for External Use’ (2018) <https://www.cqc.org.uk/sites/default/files/20181205_cqc-gmc_joint_operational_protocol_redacted.pdf> accessed 12 June 2020.

136 ibid.

137 ibid.

138 CQC and others, ‘Emerging Concerns Protocol’ (CQC 2018).

139 GDC, GMC, GPHC, HCPC, and NMC.

140 CQC and others (n 139) 6.

141 G James, ‘Report of the Independent Inquiry into the Issues raised by Paterson’ (House of Commons 2020) 186.

142 S Devaney, *Ethics for Healthcare Regulators: Enhancing Compliance with the Seven Principles of Public Life* (Manchester Centre for Regulation 2016); Baldwin and Black (n 61); Healy (n 72).

143 General Medical Council (n 58).

144 The Guardian (n 123).

145 CQC and GMC (n 136).

146 ibid 32.

147 General Medical Council (n 45).

148 Black and Baldwin (n 97) 2–22.

149 Williams and others (n 28).

150 Examples of where mainstream media have raised the issue include: The Guardian, ‘Hospital Discharge is not Rocket Science. Why are Patients Still being Failed?’ (2016) <https://www.theguardian.com/social-care-network/2016/may/16/hospital-discharge-patients-failed-ombudsmans-report> accessed 12 June 2020; The Guardian, ‘Hospitals Show “shocking” Lack of Care Discharging Vulnerable Patients’ (2015); and The Telegraph, ‘Patients sent Home from Hospital with no Advice on How to Cope, Watchdog Finds’ (2019) <https://www.telegraph.co.uk/news/2019/06/20/patients-sent-home-hospital-no-advice-cope-watchdog-finds/> accessed 12 June 2020; <https://www.theguardian.com/society/2015/jul/21/healthwatch-hospitals-discharging-vulnerable-patients-lack-of-care-> accessed 12 June 2020.

151 Parliamentary and Health Service Ombudsman, ‘Breaking Down the Barriers: Older People and Complaints about Health Care’ (PHSO 2015).

152 National Health Service (n 43).

## Notes

I am grateful to Dr Sarah Devaney, and Dr Gavin Daker-White for their insightful comments on earlier drafts of this article. I am thankful to the National Institute of Health Research for funding my research. I would also like to thank my reviewers for their constructive feedback.

I am the recipient of funding by the National Institute of Health Research (award reference NIHR-INF-0702). This work was funded by the National Institute for Health Research (NIHR) Greater Manchester Patient Safety Translational Research Centre. The views expressed are those of the author(s) and not necessarily those of the NIHR or the Department of Health and Social Care.

